# Analysis of isomeric mixtures by molecular rotational resonance spectroscopy

**DOI:** 10.1002/ansa.202300021

**Published:** 2023-06-14

**Authors:** Justin L. Neill, Luca Evangelisti, Brooks H. Pate

**Affiliations:** ^1^ BrightSpec, Inc. Charlottesville Virginia USA; ^2^ Department of Chemistry “G. Ciamician” University of Bologna Ravenna Italy; ^3^ Department of Chemistry University of Virginia Charlottesville Virginia USA

## Abstract

Recent developments in molecular rotational resonance (MRR) spectroscopy that have enabled its use as an analytical technique for the precise determination of molecular structure are reviewed. In particular, its use in the differentiation of isomeric compounds—including regioisomers, stereoisomers and isotopic variants—is discussed. When a mixture of isomers, such as resulting from a chemical reaction, is analyzed, it is highly desired to be able to unambiguously identify the structures of each of the components present, as well as quantify them, without requiring complex sample preparation or reference standards. MRR offers unique capabilities for addressing this analytical challenge, owing to two factors: its high sensitivity to a molecule's structure and its high spectral resolution, allowing mixtures to be resolved without separation of components. This review introduces core theoretical principles, an introduction to MRR instrumentation and the methods by which spectra can be interpreted with the aid of computational chemistry to correlate the observed patterns to molecular structures. Recent articles are discussed in which this technique was applied to help chemists complete challenging isomer analyses. Developments in the use of MRR for chiral analysis and in the measurement of isotopically labeled compounds are also highlighted.

**List of Abbreviations**
DFT‐D: dispersion‐corrected density functional theory
DHAA: dihydroartemisinic acid
ee: enantiomeric excess
FID: free induction decay
FWHM: full‐width at half‐maximum
GC: gas chromatography
MRR: molecular rotational resonance
MS: mass spectroscopy
NMR: nuclear magnetic resonance
TFPO: trifluoropropylene oxide

## 1 INTRODUCTION

Molecular rotational resonance (MRR) spectroscopy, also commonly known as simply rotational spectroscopy or—using the frequency range of light employed—microwave spectroscopy,^1^ is an emerging tool for identifying and confirming the structures of pure compounds as well as for performing quantitative analysis in complex mixtures by direct measurement. While instruments to measure the rotational spectra of molecules have been reported since the 1940s,^2^ these have been mainly used in fundamental research to study structural parameters of pure compounds and weakly bound complexes in the gas phase. Significant developments in two particular areas, however, have enabled the analytical applications that will be described in this Review. The first is that the measurement speed and sensitivity have improved significantly due to improvements in MRR instrument designs, not only making analytical measurements more practical but also enabling quantitative analysis of mixtures (Section 2). Meanwhile, quantum chemical methods for the accurate prediction of equilibrium geometry, from which the rotational spectroscopy parameters are derived, have improved dramatically to the point where MRR spectroscopy can identify new compounds with high confidence and without the need for a reference sample (Section 1.3).

MRR spectroscopy measures transitions between the energy levels of the rotational kinetic energy. When a molecule freely rotates, which requires producing a gas sample with low collision frequency, the rotational kinetic energy is quantized as a result of the quantization of angular momentum. For a given amount of angular momentum, the rotational kinetic energy about the molecular center‐of‐mass depends very sensitively on the shape of the molecule. Specifically, the rotational kinetic energy is related to the angular momentum via the moments‐of‐inertia in the principal axis system. For rotation about a principal axis, the moment‐of‐inertia, *I*, is

(1) $$I=\sum_{i}m_{i}R_{i}^{2}$$

where *m_i_* is the nuclear mass and *R_i_* is the distance of the *i* ^th^ nucleus from the principal axis. Therefore, molecules are distinguished based on the three‐dimensional arrangement of their atoms in space.

Although MRR spectroscopy has general applicability in analytical chemistry, the connection between the spectrum and the mass distribution makes it uniquely suited to the analysis of all types of isomeric compounds. The dependence of the spectral signature on the mass distribution distinguishes MRR spectroscopy from mass spectrometry (MS). However, it also offers complementary analysis capabilities to nuclear magnetic resonance (NMR) spectroscopy, where the nuclei‐specific nature of the spectrum can make it difficult to analyze isomers with the same functional groups, such as regioisomers and diastereomers,^3^, ^4^, ^5^ leading to a great deal of interest in correcting misassigned NMR structures.^6^, ^7^ While the rotational spectrum cannot directly differentiate enantiomers since they have the same moments‐of‐inertia, new techniques have been developed for this challenging chemical analysis. Lastly, changes in the moment‐of‐inertia, Equation (1), are also produced when the geometry of the molecule (*R_i_*) is fixed, but the masses of the nuclei (*m_i_*) change. As a result, every chemically distinct isotopic variant of a molecule has its own unique MRR spectrum. This special feature of MRR spectroscopy provides new capabilities for stable isotope analysis.

Unique features of instruments used in MRR spectroscopy also play a key role in their analytical chemistry applications. First, MRR datasets have exceptionally high spectral resolution. Therefore, MRR can directly analyze complex chemical mixtures with minimal spectral overlap. This feature of MRR measurements is essential to unlocking the potential of the technique for isomer analysis. In many cases, isomer mixtures are difficult to separate by chromatography—for example, in the separation of enantiomers using chiral columns.^8^, ^9^ In the case of isotopic mixtures, chromatographic separation is virtually impossible. The ability to distinguish isomers through their spectral signatures, and to directly analyze complex mixtures of isomers without the need for chromatographic separation, is a strength of MRR spectroscopy. Secondly, the characteristic frequencies of a molecule by MRR are determined with extraordinary accuracy due to the use of atomic frequency standards, such as 10 MHz rubidium standards, to ensure accuracy in the measured transition frequencies. Also, there is no solvent effect on the spectrum frequencies because the measurement is made on isolated molecules in the gas phase. These features combine to give MRR measurements exceptional transferability between instruments. This results in a technique that, as E. Bright Wilson Jr. described, is “a means of recognition which has absolutely no rival. Once a molecule has been caught and finger‐printed by this method, it is forever recognizable.”^10^

This Review introduces the principles of MRR theory and experimentation and highlights the capabilities of this technique for the resolution of isomers. Recently published studies, with a focus on MRR analyses that are difficult for other analytical techniques, will be reviewed. New capabilities in enantiomeric analysis, as well as in the resolution of isotopic mixtures, will receive particular attention due to gaps in currently available analytical techniques. Finally, we will conclude with a brief discussion of some of the remaining developmental challenges for MRR and an outlook for the technique.

### 1.1 Theoretical basis of the rotational spectrum

The quantized rotational kinetic energy levels of a freely rotating, rigid, nonlinear molecule are determined by solving the rigid‐rotor Hamiltonian:^1^

(2) $${\hat{H}}_{rot}=A{\hat{P}}_{a}^{2}+B{\hat{P}}_{b}^{2}+C{\hat{P}}_{c}^{2}$$

where *A*, *B* and *C*, the rotational constants, are defined by

(3) $$=\frac{h^{2}}{8\pi^{2}I_{a}},\;B=\frac{h^{2}}{8\pi^{2}I_{b}},\;C=\frac{h^{2}}{8\pi^{2}I_{c}}$$

*I*_a_, *I*_b_ and *I*_c_ are the moments‐of‐inertia about the three principal axes of the molecule as defined in Equation (1). The operators in the Hamiltonian of Equation (2) are the angular momentum operators for rotation about each of the principal axes (*a, b, c*). The axes are defined such that *I*_a_ ≤ *I*_b_ ≤ *I*_c_. Some molecules have symmetry such that two of these axis have identical moments of inertia, such as CH_3_CN, and are called symmetric tops. Meanwhile, for linear molecules such as HCN, the *I*_a_ moment of inertia is essentially zero (since all of the mass lies along this axis). These special cases have simple formulas for their allowed energy levels, and are often covered in physical chemistry texts. Asymmetric top molecules, with three distinct principal moments‐of‐inertia, do not have simple formulas for their energy levels, but the process of solving the molecular Hamiltonian from Equation (2) using computer programs is nevertheless straightforward and exact. Additionally, as the moments‐of‐inertia can be easily calculated for any given equilibrium molecular geometry, it is simple to forward‐predict the MRR spectrum for a molecule from this information.

MRR transitions are measured through the interaction of a sample with a microwave or millimeter‐wave electric field with a frequency component corresponding to the difference between energy levels, through the relationship Δ*E* = *hν*. The allowed transitions follow a set of well‐defined selection rules, allowing only certain changes in the angular momentum quantum numbers. The transition moment is proportional to the molecule's permanent dipole moment projected along one of the three principal axes (*μ*_a_, *μ*_b_, or *μ*_c_):with transitions characterized as being either *a*‐type, *b*‐type, or *c*‐type.

MRR analyses sometimes require additional parameters in the Hamiltonian beyond Equation (2), such as to account for centrifugal distortion, nuclear quadrupole coupling (with the most common nuclei being^14^N,^35^Cl/^37^Cl and^79^Br/^81^Br):and internal angular momentum that can be generated, for example, by low‐barrier rotation of a methyl group. However, in practice the spectra of most compounds in the microwave region can be characterized well using the three rotational constants, with the other terms providing relatively small adjustments. A number of programs have been developed to allow the prediction and analysis of rotational spectra, most notably the SPFIT/SPCAT package developed at the Jet Propulsion Laboratory,^11^ but others have been developed by different research groups, including with graphical user interfaces.^12^, ^13^

The intensities of rotational transitions depend not only on the dipole moment components as described above but also on the population difference between the two energy levels. This population difference depends on the rotational partition function (*Q*_rot_) and the rotational temperature (*T*_rot_). The rotational partition function gives an estimate of the number of rotational energy levels with a significant population, while the rotational temperature determines the distribution of the molecular sample across the available energy levels. The rotational partition function, for a nonlinear molecule, depends on the rotational temperature, and on the rotational constants, according to the following relationship:^1^

(4) $$Q_{\mathrm{rot}}\propto \sqrt{\frac{{T_{\mathrm{rot}}}^{3}}{A\times B\times C}}$$

One consideration for rotational spectroscopy measurements is that low temperatures will increase the population differences and transition strength. As a result, instruments using pulsed jet expansions that lower the rotational temperature of the gas sample by adiabatic expansion are common. Furthermore, for a fixed temperature, the rotational partition function increases with molecular size since the rotational constants decrease as the molecular weight increases. This leads to a rotational spectrum that contains more lines for analysis, but each line is correspondingly weaker. As an example, the lowest‐energy conformational isomer of menthol (C_10_H_20_O, molecular weight 156 g/mol in the most abundant isotopic species) has rotational constants approximately *A* ∼ 1779.8 MHz, *B* ∼ 692.6 MHz, *C* ∼ 573.3 MHz,^14^ resulting in a partition function of about 200 at a rotational temperature of 1 K (a rotational temperature typically achieved with a pulsed supersonic expansion nozzle). For a molecule at about double this mass, the methyl ether of Frondosin B (C_21_H_26_O_2_, molecular weight 310 g/mol in the most abundant isotopic species) the rotational constants are approximately *A* ∼ 433.4 MHz, *B* ∼ 190.5 MHz, *C* ∼ 145.7 MHz.^15^ This results in a partition function of approximately 1532, a 7.6‐fold increase, and a corresponding decrease in the sensitivity of the spectrum of the larger molecule.

### 1.2 Sampling in MRR spectroscopy

Rotational spectroscopy measurements can be performed on any gas‐phase sample where the pressure is low enough for molecules to undergo unhindered rotation, including room‐temperature, low‐pressure gas cells^16^, ^17^, ^18^ and—in a special extreme case—cold gas clouds in interstellar space.^19^, ^20^, ^21^ However, in order to measure analytes above ∼150 amu, it is generally necessary to rotationally and vibrationally cool the sample before analysis. The vibrational partition function for a sample at room temperature increases rapidly with size due to the strong effect of low‐frequency vibrational modes. As each vibrational state has a distinct rotational spectrum, this dilution rapidly makes the detection of large molecules by MRR spectroscopy unfeasible at room temperature.

The most common method of achieving effective sample cooling is by the use of a pulsed supersonic expansion valve and a widely used, robust device is the Parker Series 9 high‐speed valve. These valves achieve analyte rotational temperatures of 1–2 K and provide near‐complete vibrational cooling as well as some conformational relaxation.^22^, ^23^ The sample path in the valve body can be held at high temperature (up to approximately 250°C), preventing large molecular weight analytes from condensing before they can be injected into the chamber for analysis—without affecting the rotational temperature of the expansion. A noble carrier gas is used, with neon generally giving the best sensitivity but helium and argon also in common use. The supersonic expansion can also stabilize non‐covalent molecular complexes, a capability that is employed for chiral analysis through chiral tagging as will be described later in this review.

Alternative methods for preparing cold samples for analysis are in use including continuous jets^24^, ^25^ and a buffer gas cooling method.^26^ These techniques also can achieve low rotational and vibrational temperatures (below 10 K), and have the beneficial feature that measurements can be performed continuously, by contrast, typical pulsed supersonic expansion measurements are limited to between 3 and 10 Hz, depending on the speed of the pumping system. However, these methods can require more sample to reach the same detection limits. In the remainder of this article, the spectra shown and discussed were acquired using MRR spectrometers with pulsed supersonic expansion valves.

The volatilization of samples is also a critical concern in MRR. The analyte concentration at which sensitivity is optimized in a pulsed jet expansion has been found to be approximately 0.1%. With a typical gas backing pressure of 1–2 bar, the analyte partial vapour pressure should be in the range of 1‐2 mbar (0.75–1.5 Torr). For analytes with vapor pressures in this range at a temperature below 250°C, the most common method is to heat the sample to generate enough vapor for analysis, which is then entrained in the carrier gas stream and injected into the spectrometer. For molecules with lower vapor pressures, or that thermally degrade before volatilization, one approach that is demonstrated is laser ablation.^27^, ^28^ In this method, the analyte is pressed into a rod and irradiated with a pulsed laser to produce volatilized analytes, which are then swept in the carrier gas. Laser ablation sampling interfaces have allowed for the analysis of biologically relevant molecules including amino acids,^29^ dipeptides,^30^ and sugars.^31^ As will be discussed in the last section of this review, further development is needed on effective and robust sampling methods for MRR.

### 1.3 Identification of compounds with MRR spectroscopy

Identification of a previously unmeasured molecule with MRR spectroscopy, in most cases, relies on the agreement between the experimentally derived rotational constants and those calculated for a proposed structure. To use MRR as a structural confirmation or elucidation tool, computational chemistry methods that can predict the molecular mass distribution accurately are required. Especially as the analyte size increases, long‐range dispersive interactions become more important in defining the geometry, as such, accurate calculation of their effect is critical for using MRR to identify new molecules. The recent successes of MRR spectroscopy in analytical chemistry rest equally on instrument development and improved quantum chemistry methods.

The density functional theory methods with empirical dispersion corrections (DFT‐D) developed by the Grimme research group^32^, ^33^, ^34^ have been demonstrated over the past decade to provide accurate predictions of MRR spectral parameters, with modest computational resource requirements. In particular, the B3LYP‐D3BJ functional, with a 6‐311++G(d,p) basis set, is found to reproduce molecular rotational constants to within 2%, even when long‐range interactions are present, and generally predicts the dipole moment direction, nuclear quadrupole hyperfine coupling (when present), and relative conformational energies with good accuracy. For molecules with multiple low‐energy conformational isomers, an automated computational search is employed first to find all of the possible minima, followed by geometry optimization with DFT‐D methods to determine the MRR parameters and relative energies for each conformer. Each conformer is detected as an independent pattern in the spectrum, as the structure is frozen for the duration of the MRR experiment (<100 µs).

MRR spectroscopy can also be used to determine molecular structures more directly through the measurement of isotopologues in natural abundance. Using a method first published by Kraitchman, the magnitude coordinates of an atom in the molecule's principal axis system can be derived from the change in its moments of inertia with isotopic substitution.^35^, ^36^ So it is possible to determine the heavy (i.e., non‐hydrogen) atom skeleton through the minor isotopologues naturally present at low abundance in the sample. In practice, this refers to ^13^C,^15^N and ^18^O, which are at approximately 1.1%, 0.36% and 0.21%, respectively, relative to the most abundant isotopes. This has been utilized to give accurate structural information for a wide variety of molecules.^37^, ^38^, ^39^, ^40^ However, this requires spectra with high enough sensitivity to detect these minor isotopologues, which can require long measurement runs and large sample quantities. For most analytical applications, such as those described later in this Review, the agreement between experimental and computed structural parameters is sufficient to enable the molecular assignment.

## 2 INSTRUMENT IMPLEMENTATIONS

### 2.1 Broadband MRR spectrometer

**FIGURE 1:**
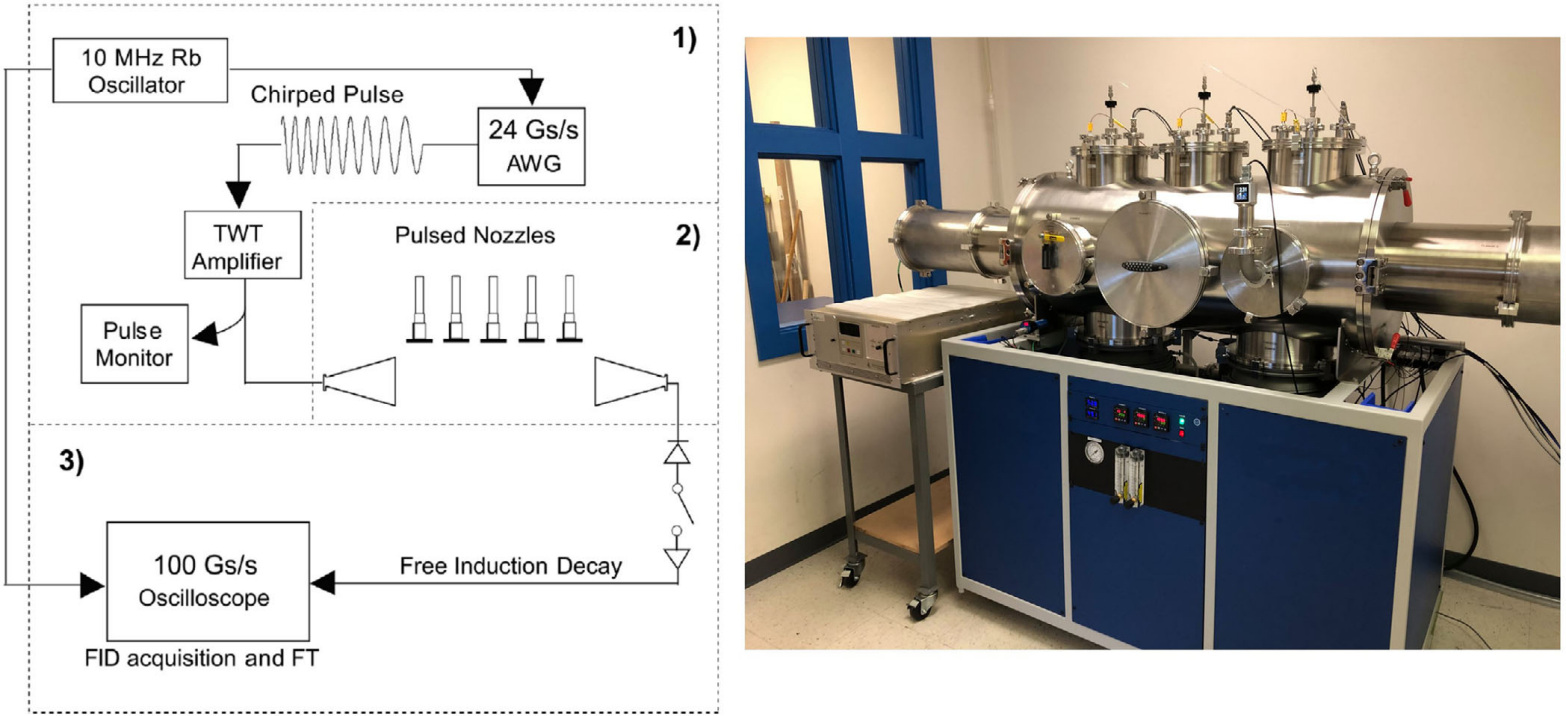
Implementation of chirped‐pulse broadband molecular rotational resonance (MRR) spectroscopy for chemical analysis. The left panel shows a schematic of a direct‐digital spectrometer with multiple pulsed nozzle sources (used with permission ^43^), with the three aspects of the instrument, specifically pulse generation (1): excitation (2) and detection (3), indicated. The right panel shows a photograph of a broadband MRR spectrometer. This instrument implementation has a total lab footprint of approximately 144 × 36 in (365 × 90 cm).

In recent years, major advances in MRR spectroscopy instrumentation have been made to enable its use in analytical chemistry applications. The development of broadband Fourier transform MRR spectrometers with chirped‐pulse excitation enabled the simultaneous measurement of a large range of the spectrum to allow for efficient characterization of new compounds and mixtures.^41^ A schematic of the operating instrument is shown in Figure 1, as well as a photograph of a commercial broadband MRR spectrometer.

The design and operation of the broadband MRR spectrometer will only be briefly described here, as it is detailed in other publications.^42^, ^43^, ^44^ After a molecular sample is injected into the spectrometer, it is excited by a chirped microwave pulse (a linear frequency sweep) with a typical duration of 1–4 µs. This chirped pulse is generated using an arbitrary waveform generator and amplified using a high‐power microwave amplifier, most commonly a traveling wave tube amplifier. A broadband microwave antenna broadcasts this excitation pulse into the chamber to interact with the molecular sample, which creates a macroscopic polarization of the dipole moments of the molecular ensemble, and a coherent emission signal at the resonance frequency of each allowed rotational transition. A second horn antenna serves as the receiver for this emission. A diode limiter and/or PIN diode switch are used to protect the receiver from damage due to the high‐power pulse. Following these protection electronics, the coherent emission is amplified and digitized using a digital oscilloscope. As in NMR spectroscopy, the coherent emission lasts for a finite time due to dephasing. The free induction decay (FID) in MRR spectroscopy is caused by residual Doppler broadening in the pulsed jet sample. The rotational spectrum is finally obtained by fast Fourier transformation of the coherent emission, averaged over a user‐specified number of measurement repetitions. Because the peak of the molecular pulse lasts for several hundred microseconds, as compared to typical FID relaxation times of 20‐40 µs, it is common to perform multiple excitation measurements on each gas pulse. The typical full‐width at half maximum (FWHM) for a transition in a chirped‐pulse MRR spectrometer, after typical processing with a Kaiser‐Bessel apodization window for sidelobe suppression,^42^ is on the order of 70 kHz. Compared to a typical measurement bandwidth of ∼6–10 GHz, these instruments can detect thousands of transitions simultaneously with few, if any, spectral overlaps.

In the original implementations of the chirped‐pulse MRR spectrometer, a full spectral band (with the most common ranges being 2–8 and 6–18 GHz) is excited on each gas pulse. In these spectrometers, with the use of high‐gain horn antennas, the horns can be positioned at a distance such that multiple nozzle sources are placed between the two horns. The molecular sample is divided between the nozzles. By pulsing the nozzles simultaneously, the signals resulting from each nozzle add coherently, which reduces the measurement time required to achieve the target sensitivity by *N* ^2^ and the sample consumption by a factor of *N* (where *N* is the number of nozzles used).^42^ MRR spectroscopy with smaller vacuum chambers, lower‐power amplifiers and/or lower simultaneous measurement bandwidths have also been developed for different research applications.^45^, ^46^, ^47^

### 2.2 Cavity‐enhanced MRR spectrometer

Prior to the design of the chirped‐pulse Fourier‐transform microwave spectrometer, the main instrument for pulsed‐jet MRR spectroscopy was the cavity‐enhanced Fourier transform spectrometer. Introduced by Balle and Flygare,^48^ this instrument coupled the pulsed supersonic expansion with a high‐finesse microwave cavity that provides passive amplification of the polarization pulse and the molecular emission. Using high‐reflectance mirrors, it gives a cavity with a quality factor (*Q*) in the 10^3^‐10^5^ range. One of the mirrors is mounted on a translation stage to allow the resonant frequencies of the cavity to be tuned, however, the bandwidth of a single measurement is limited to a few MHz at most by the cavity. Therefore, this instrument requires a large number of measurements to fully characterize a new molecular system. This approach results in long measurement times and high sample consumption—measurement issues solved by the chirped‐pulse spectrometer design. Instead, the narrow‐band, cavity‐enhanced performance is well‐suited to targeted measurements where the rotational frequencies for the molecule(s) of interest are already known. In fact, to measure an already known molecular transition, as compared to the broadband spectrometer, the cavity‐based MRR spectrometer requires less time to reach the same signal‐to‐noise ratio and consequently consumes much less sample by about a factor of 100, enabling practical analyses with 1 mg or less of sample.

Over the past few decades, a number of research groups have designed and built different versions of the cavity‐enhanced Fourier transform rotational spectrometer. Some of the most notable include an instrument with cryogenically cooled mirrors to enhance sensitivity;^49^ versions with larger mirrors to reach very low frequency;^50^, ^51^ and a smaller vacuum chamber to enable a portable instrument.^52^ This smaller design, developed at the National Institute of Standards and Technology (USA) in the 1990s, served as the model for a commercial targeted MRR product line developed at BrightSpec. In this design, the entire instrument—vacuum chamber, backing pumps, measurement electronics, sampling and the control computer and workstation—were mounted on a single cart with dimensions of 36 × 24 in (approx. 90 × 60 cm) that could be readily moved between laboratories. Some research groups have also built spectrometers capable of measuring both chirped‐pulse and cavity‐enhanced spectra on the same vacuum chamber and sample source, to gain the advantages of both instrument designs.^53^

## 3 EXAMPLES OF ISOMERIC IDENTIFICATION AND QUANTIFICATION USING MRR

The combination of high spectral resolution and structural specificity in MRR spectroscopy brings unique capabilities for the quantitative analysis of isomeric compounds in mixtures. This section will highlight recent studies where this is applied in different ways to support the understanding of chemical reactions and processes. Since MRR spectroscopy has only recently been used in analytical chemistry, we first present an example spectrum analysis to give a better understanding of the technique.

### 3.1 An example MRR broadband spectrum analysis

**FIGURE 2:**
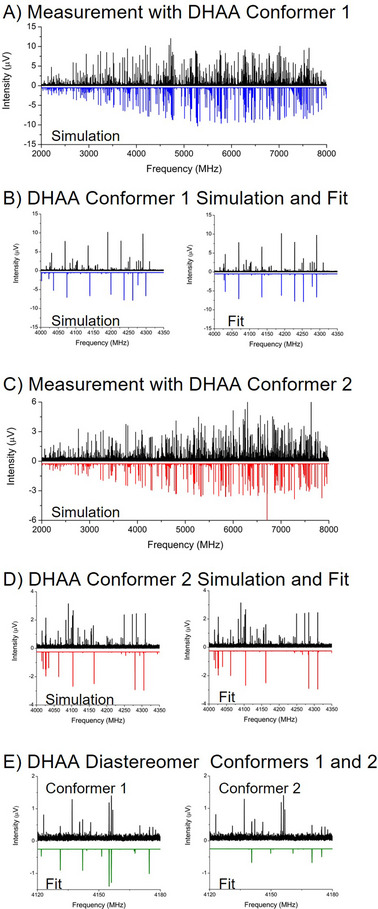
Progression of analysis of a mixture of diastereomers using broadband molecular rotational resonance (MRR) spectroscopy. In each panel, the black spectrum indicates the experimental spectrum, while the different colours are predicted spectra using calculations of the rotational Hamiltonian (Equation (2)) with a given set of rotational constants, and are negative‐going for visualization. Panels marked “Simulation” use computed rotational constants, while “Fit” spectra use experimentally determined parameters. See the text for further description.

The MRR measurement principles described in the previous sections are illustrated with the analysis of a reaction flask sample of dihydroartemisinic acid (DHAA).^54^ The sample is prepared through the hydrogenation of one of the double bonds in artemisinic acid—the first reaction step in the preparation of the antimalarial drug artemisinin.^55^ The crude reaction product mixture also contains starting material and over‐reduction impurities (where both double bonds in artemisinic acid are reduced). A key analysis goal is determining the ratio of the desired DHAA reaction product and its diastereomeric impurity, since subsequent purification by crystallization requires this impurity to be present at less than about 15% of the target reaction product. The MRR spectrum of the sample is shown in Figure 2A. This was performed by measuring 50 mg of the crude oil product in a 2–8 GHz chirped‐pulse Fourier transform microwave spectrometer. The sample was heated to 160°C and 350,000 time‐domain averages of the FID were acquired.

**FIGURE 3:**
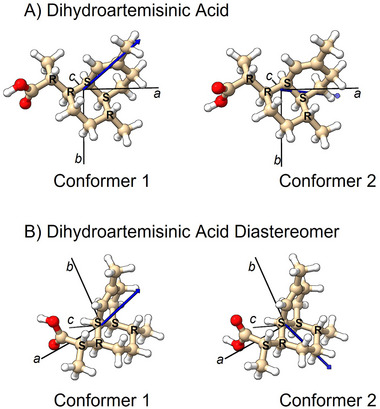
Computed structures of dihydroartemisinic acid (DHAA) and its diastereomeric impurity observed in the hydrogenation of artemisinic acid as described in the text. Each of these structures is identified in the molecular rotational resonance (MRR) spectrum through the agreement of its calculated rotational constants with experimentally determined values (Table 1). The principal axis system (black lines) and the direction of the permanent dipole moment (blue arrows) are also indicated on the structures.

Unlike spectroscopic techniques in analytical chemistry that have transition frequencies dependent primarily on the nuclear environment (in NMR spectroscopy) or bonds (in infrared spectroscopy), there are no empirical rules for “spectrum interpretation” in MRR spectroscopy. Instead, each distinct molecular geometry will produce a spectral pattern that depends on the mass distribution about the center‐of‐mass, characterized by the rotational constants of Equation (3), and the direction of the dipole moment vector. The analysis proceeds by calculating the equilibrium geometries of candidate reaction products using quantum chemistry geometry optimization. The blue spectrum in Figure 2A, plotted in a negative‐going direction for comparison to the experimental spectrum, shows the calculated MRR spectral signature of the lowest energy conformer of the desired reaction product, DHAA. The quantum chemistry equilibrium geometry of this species is shown in Figure 3A. The structures in this study were calculated using Gaussian 09.^56^

An expanded view of the spectrum is shown in Figure 2B, where the left panel shows the comparison between the theoretical (blue) and measured (black) spectra. The process of fitting an MRR spectrum involves the assignment of observed transition frequencies to the corresponding quantum number transitions derived from the simulation. The rotational constant parameters of the Hamiltonian, Equation (2), are adjusted in a least‐squares fitting process to determine new values that now predict the experimental transition frequencies. New transitions are progressively added to the fit and the constants are further refined until a final set of experimental rotational constants is determined. In a microwave spectral analysis, the number of transition frequencies fits is always much greater than the number of parameters required, leading to a large amount of redundancy and leaving no chance of a coincidental set of transitions. In the case of DHAA, a total of 412 rotational transitions in the spectral pattern are observed and used to fit the rotational constants. The root‐mean‐squared frequency error in the fit is 9 kHz—a small fraction of the experimental linewidth (70 kHz FWHM). This fit was achieved using only five parameters—the three rotational constants as well as two centrifugal distortion constants to account for slight deformation of the molecule as the rotational angular momentum increases.

The fit spectrum is shown for DHAA in the right panel of Figure 2B, and the experimental and fit rotational constants are summarized in Table 1. The close match between the experimental and theoretical rotational constants, with differences below 1%, shows an excellent match to the structure. Although the spectrum in Figure 2A appears dense, it is important to note that the 412 assigned transitions in the MRR spectral pattern only occupy about 30 MHz of total spectral bandwidth—compared to the 6000 MHz frequency bandwidth of the measurement. As a result, once the MRR spectrum of a species is fit, the spectrum can be removed from the analysis by simply cutting the observed transitions from the data set.

**TABLE 1:** Experimental and theoretical rotational constants for dihydroartemisinic acid and its diastereomer impurity.

A. Dihydroartemisinic Acid						
	Conformer 1			Conformer 2		
	Experiment	Theory^a^	%Error	Experiment	Theory^a^	%Error
*A* (MHz)	717.52200(12)	714.75	–0.39	715.13265(12)	713.56	–0.22
*B* (MHz)	311.494388(57)	311.73	0.08	312.860580(74)	313.11	0.08
*C* (MHz)	254.837729(63)	255.45	0.24	258.630310(88)	258.34	–0.11
*Δ*_J_ (Hz)^b^	4.63(21)			4.38(29)		
*Δ*_JK_ (Hz)^b^	16.35(94)					
*μ*_a_ (D)		1.1			1.6	
*μ*_b_ (D)		–1.1			0.4	
*μ*_c_ (D)		–0.7			1.4	
N^c^	412			226		
σ (kHz)^d^	8.9			7.2		

B. Diastereomer Impurity of Dihydroartemisinic Acid						
	Conformer 1			Conformer 2		
	Experiment	Theory^e^	%Error	Experiment	Theory^e^	%Error
*A* (MHz)	631.38942(56)	630.18	–0.19	632.06923(58)	631.09	–0.15
*B* (MHz)	360.81357(20)	360.07	–0.21	361.98926(17)	360.49	–0.41
*C* (MHz)	283.69638(31)	283.22	–0.17	285.05402(20)	284.66	–0.14
*Δ*_J_ (Hz)	25.3(31)			6.37(84)		
*Δ*_JK_ (Hz)	–8.7(50)			34.0(80)		
*μ*_a_ (D)		–0.8			–0.8	
*μ*_b_ (D)		0.5			–1.5	
*μ*_c_ (D)		–1.3			0.3	
N	170			87		
σ (kHz)	14.1			8.4		

^a^ Quantum chemistry geometry optimizations were performed using B2PLYPD3/6‐311++G** in Gaussian 09.

^b^ *Δ*_J_ and *Δ*_JK_ refer to quartic centrifugal distortion constants in Watson's A‐reduction.^93^

^c^ Number of independent transitions used in the spectroscopic analysis.

^d^ Root‐mean‐square difference between experimentally observed and calculated line frequencies.

^e^ Quantum chemistry geometry optimizations were performed using the B2PLYPD3/6‐311++G** in Gaussian 09.

The experimental spectrum with the lowest energy conformer of DHAA cut from the data set is shown in Figure 2C. The red simulated spectrum is the theoretical MRR spectrum for the second lowest energy conformer of DHAA shown in Figure 3A. This conformer has a 180^o^ dihedral angle change in the carboxylic acid group. Although this is a small change in the overall geometry, this conformer gives a distinct MRR spectrum. The expanded scale views of the simulated and fit MRR spectra for this conformer are shown in Figure 2D, and the rotational constants are also reported in Table 1. The rotational constants for the two conformers of the desired DHAA reaction product are similar, suggesting that definitive attribution of the experimental spectra to specific conformers could be difficult. However, the dipole moment direction, conveyed by giving the vector components of the dipole moment in the principal axis system, differs between the two isomers so that the intensity profile of the spectrum can be used to differentiate them (although the details of this analysis are not presented here). A key point of this analysis is that a single chemical species may have multiple conformational isomers populated in the pulsed jet gas sample, which need to be analyzed separately so that the total amount of the substance can be determined.

The important impurity in the DHAA synthesis is the diastereomer that can be produced when the new chiral centre near the carboxylic acid group is created by the reduction of the double bond. The equilibrium geometries of the two low‐energy conformers of this diastereomer impurity are shown in Figure 3B. This impurity is easily identified in the experimental spectrum, with the comparison of the fit spectra and the experiment shown in Figure 2E. The comparison between experimental and theoretical rotational constants of the conformers of this impurity is also given in Table 1. As in the DHAA analysis, there is a significant difference in the dipole moment vector direction between the two conformers, so they can be identified with high confidence even though their rotational constants are similar. Note that the difference in the rotational constants for the DHAA diastereomers is much larger than the differences between the experimental and theoretical rotational constants. As a result, unambiguous identification of the diastereomer geometry is easily achieved.

Finally, we emphasize the important feature of transferability in MRR spectroscopy discussed in the Introduction. Once the spectrum of a species is assigned, it can be added to a database and the transition frequencies can be transferred to any other MRR spectrometer for identification of the molecule. Further, because the spectral resolution of MRR spectrometers is so high, there is a low probability of overlapping transitions even for complex sample mixtures. (And, given the fact that the spectral pattern has many transition frequencies, there is flexibility in finding a transition that has no spectral overlap for any given mixture.) In practice, a single transition of the MRR spectrum is diagnostic for the chemical species and can be monitored at high sensitivity using cavity‐enhanced MRR spectroscopy. This capability of MRR spectroscopy is used in a reaction monitoring application described later.

### 3.2 Identification and quantitation of regioisomers and diastereomers

MRR can aid in the characterization of new chemical reactions, especially where isomeric products can result. In one recent study, two reactions were chosen that were known to contain multiple regioisomeric products.^15^ These impurities were originally identified by scaling up the reaction to a quantity where they could be isolated and their structures elucidated by a combination of high‐resolution mass spectrometry and NMR spectroscopy. Due to this labour‐intensive process, it was desired to evaluate a method that could identify and quantify the reaction components directly from a crude mixture.

For each reaction, approximately 50 mg of the unpurified mixture was measured using a chirped‐pulse broadband spectrometer in the 2–8 GHz region over the course of approximately 3 h. Using knowledge of the reaction chemistry and the products that could result (including both those that were previously known as well as additional candidates), the computational procedure described in the previous section was used to determine the structures of the low‐energy conformers and their calculated MRR parameters. These parameters could be used to assign each species in the mixture spectrum.

**FIGURE 4:**
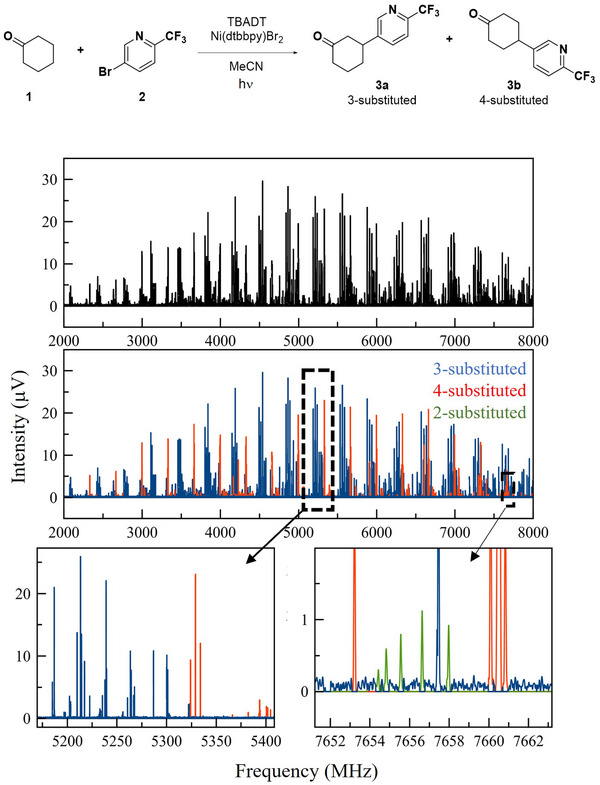
Analysis of a crude reaction product mixture by molecular rotational resonance (MRR). The reaction studied (top) is the photocatalytic arylation of C‐H bonds in cyclohexanone as described ^57^. In the top panel, the broadband MRR spectrum of the crude reaction mixture is shown. The middle and lower panels colour the transitions to indicate the species identifications, with expanded views to show the resolution of the spectrum and the identification of the low‐abundance 2‐substituted impurity. The figure is adapted with permission.^15^

An example of one of these analyses is shown in Figure 4. The reaction studied employed a novel photocatalytic methodology to directly arylate aliphatic C‐H bonds,^57^ in this case of cyclohexanone. This chemistry often resulted in a mixture of regioisomeric products. The patterns corresponding to the low‐energy conformers of the two main products (3‐substituted and 4‐substituted) could be readily identified. In addition, a much weaker pattern was detected that was assigned as the 2‐substituted isomer. This minor product was not identified in the original study of this chemistry but was later confirmed in high‐performance liquid chromatography data. In addition, a reaction byproduct where the solvent was arylated was identified from the MRR reaction mixture.

The distribution of the regioisomeric products was also quantified, using ab initio dipole moment information to calculate the response factor for each analyte. In each case, the relative quantity of each observed product was compared to that determined by chromatographic analysis. For each species, the contributions of the different observed conformational isomers were summed to give the total species abundance. MRR was found to give good agreement with the chromatographic methods within this error.

**FIGURE 5:**
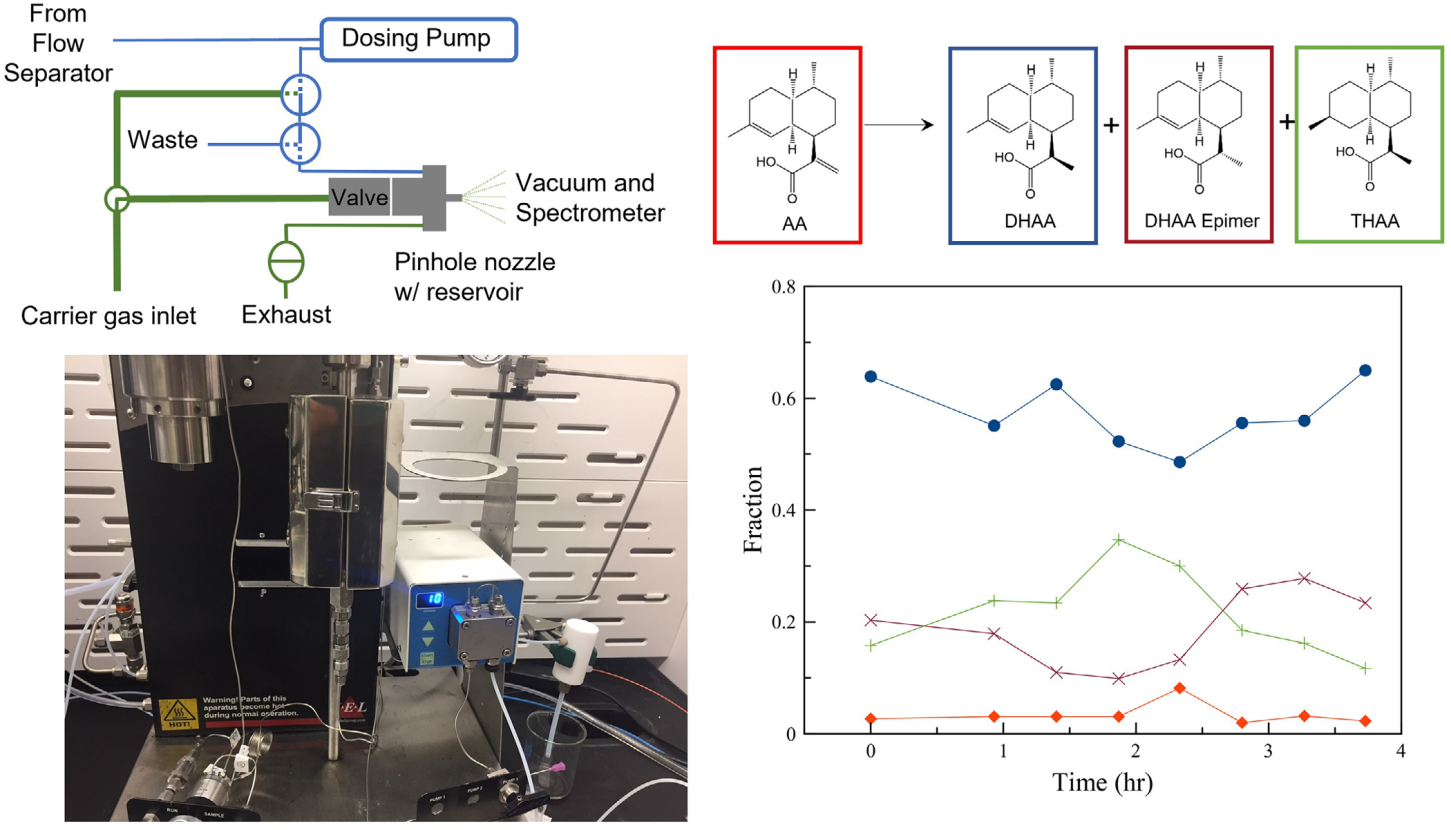
A demonstration of the use of molecular rotational resonance (MRR) for automated analysis of the output of a continuous flow reactor is presented. The solution from the reactor is transferred using a solenoid dosing pump into a reservoir in the pulsed valve. The solvent is first vented off to exhaust, in this case, the solvent was ethanol and was vented at 75°C. Following this, the temperature of the valve is increased to 160°C and the analyte mixture is volatilized and measured. The reaction studied was the asymmetric hydrogenation of artemisinic acid (top right). The relative concentrations of the four species found in the reaction mixture are quantified on a ∼25‐min timescale. Figure adapted with permission.^54^

For each species identified in a broadband MRR measurement, the fit rotational constants and transition frequencies can be used to allow faster subsequent analyses on a targeted MRR spectrometer. This can be utilized to enable reactions performed under different conditions to be compared for reaction optimization. The ability of targeted MRR measurements to quantify impurities, including isomers, by online coupling to a continuous reaction system has also been evaluated (Figure 5).^54^ The reaction in this study was the asymmetric hydrogenation of artemisinic acid that was discussed above. A sampling interface was designed to periodically pull an aliquot of fresh reaction solution from the process, volatilize the solvent, and then increase the interface temperature to volatilize the compounds of interest. The product yield, diastereoselectivity, and level of a byproduct resulting from the unwanted hydrogenation of another double bond in the molecule could all be determined in a single, automated measurement. The results were also compared to ^1^H NMR analyses, with good quantitative agreement between the two techniques and MRR enabling a much faster time from sample withdrawal to measurement results (25 min for MRR, as opposed to ∼4 h for ^1^H NMR). In each of these studies, representative samples and/or standards were first measured on a broadband spectrometer, leading to the determination of experimental transition frequencies that enabled these rapid targeted analyses.

Another application for targeted MRR measurements is to verify the levels of undesired impurities in starting materials. As an example, the detection and quantification of impurities in 2,4‐difluorobenzylamine, which is a starting material used in the synthesis of the human immunodeficiency virus drug cabotegravir, was studied.^58^ The quantitation of impurities with fluorine atoms in different positions of this starting material is crucial to ensuring the final drug will meet purity requirements. This can include mono‐ and unfluorinated variants which, while not of equal molecular weight, are nevertheless difficult to separate chromatographically from the main compound.^59^ In this study, the ability of MRR to rapidly and accurately quantify four possible impurities in 2,4‐difluorobenzylamine samples was demonstrated, with detection limits <0.05% and excellent linearity (*R* ^2^ > 0.999) in a rapid measurement (200 s of measurement time per impurity, or less than 20 min for the entire mixture including cleaning time between samples).

### 3.3 Chiral analysis by MRR

Enantiomer pairs, as exact mirror image molecules, have the same moments‐of‐inertia and so the same transition frequencies in an MRR spectrometer. Therefore, as for other spectroscopic methods, an asymmetric aspect must be added to the measurement to distinguish them. Two approaches have been demonstrated for this. The first to be reported, called three‐wave mixing,^60^, ^61^, ^62^ exploits a subtle feature of chirality. For a chiral molecule, the sign of the products of the dipole moment vector components in the principal axis system of molecular rotation is opposite. Three‐wave mixing rotational spectroscopy provides a measurement scheme that produces a signal that depends on the dipole moment vector component product. It utilizes two microwave excitation fields, applied at orthogonal polarization, and designed to be resonant with two MRR transitions sharing a quantum state and with different dipole moment selection rules (one *a*‐type and one *b*‐type transition, for example). Following this excitation, the sample will emit a coherent FID signal at a polarization orthogonal to the two excitation fields, and at a third frequency which is either at the sum or difference frequency of the two pulses (depending on the rotational levels involved). In this experiment, enantiomers emit FID signals with opposite phase, while a racemic mixture will produce no signal. We direct the reader to a recent review for further discussion of this technique.^63^ However, the three‐wave mixing technique has not yet, to date, established a robust experimental method to determine the absolute phase of the chiral signal. Without this instrument capability, it is not possible to determine the absolute configuration of a sample with unknown configuration.

The second approach, chiral tagging, uses a strategy similar to chiral derivatization methods and auxiliaries developed for NMR.^64^, ^65^ The MRR chiral tag approach converts the enantiomers of a molecule into diastereomers (which have distinguishable MRR spectra) through the noncovalent attachment of a small, chiral molecule of known enantiomeric composition.^66^, ^67^, ^68^ Pulsed supersonic expansion nozzles are ideal for this application, as they are well known to stabilize weakly bound complexes and allow for conformational/isomeric cooling into a small number of geometries (as described in the Introduction). Structural studies of chiral complexes in the gas phase have been performed for more than a decade, to explore chiral recognition effects.^69^, ^70^ Critically, it has been demonstrated on a number of chiral complexes that DFT‐D methods can calculate the molecular moments‐of‐inertia of weakly bound complexes, and accurately determine the energetically preferred complex geometries, with sufficient accuracy to allow the unambiguous assignment of absolute configuration on chiral compounds without reference standards. This provides a valuable, fully orthogonal complement to other techniques for absolute configuration assignment—in particular, chiroptical spectroscopies such as electronic circular dichroism,^71^ vibrational optical activity,^72^, ^73^, ^74^ and X‐ray diffraction.^75^

**FIGURE 6:**
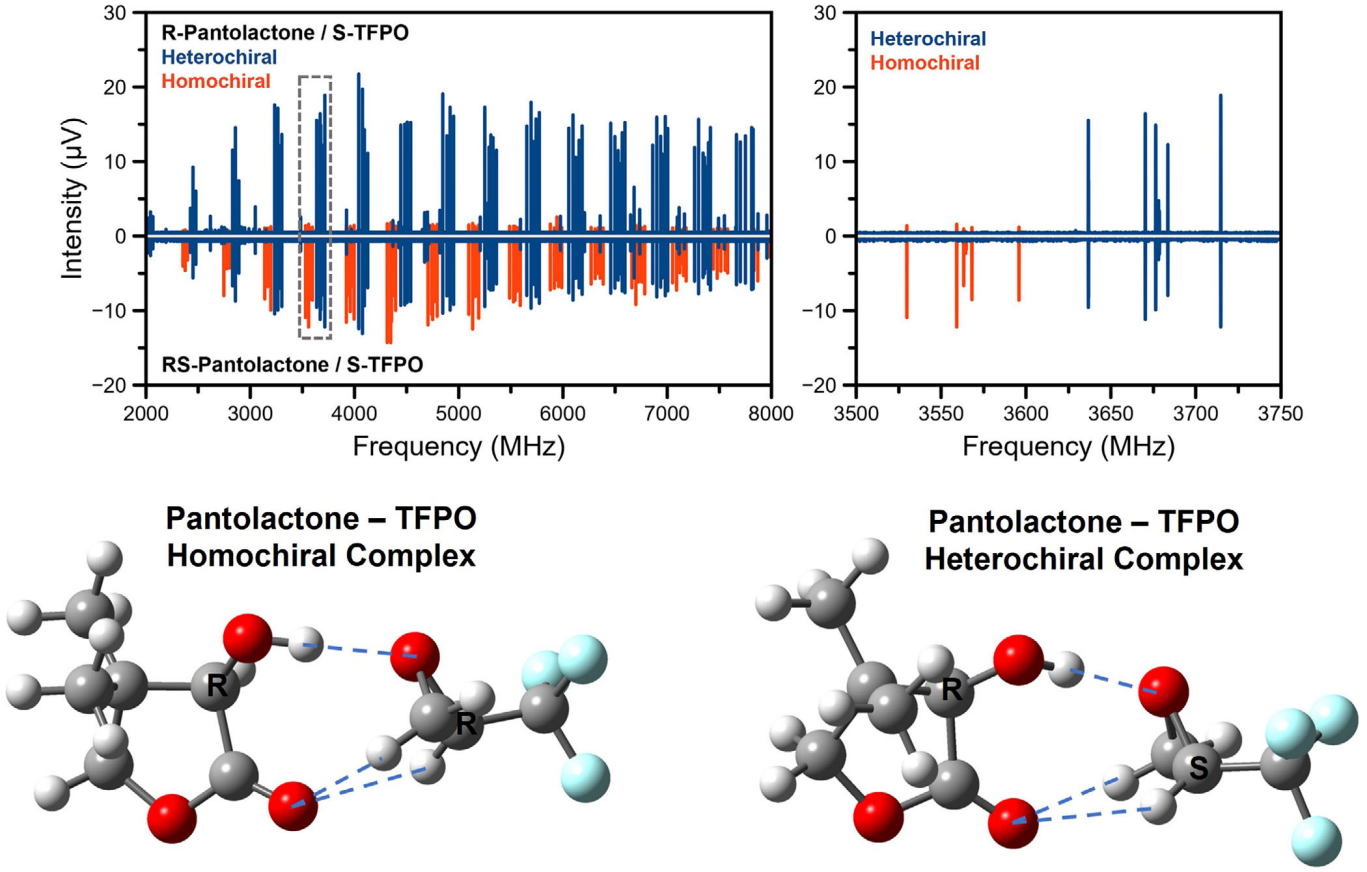
An example of the assignment of absolute configuration by chiral tagging molecular rotational resonance (MRR). In the top panel, the spectra of the chiral complexes between pantolactone and trifluoropropylene oxide (TFPO) are shown. The MRR spectra of pure pantolactone and pure TFPO have been removed from this spectrum. The negative‐going spectrum is the racemic measurement (where the two diastereomeric complexes are formed at equal concentration) while the positive‐going spectrum is where the enantiopure tag is used. The homochiral and heterochiral complexes are assigned using the agreement of the rotational constants of the calculated minimum complex geometries (bottom panels) with the experimentally derived values. In this measurement, the pantolactone absolute configuration is assigned as (R). Adapted with permission.^67^

An example of absolute configuration assignment by chiral tagging MRR is presented in Figure 6.^67^ The analyte is pantolactone, a key chiral intermediate in the production of (*R*)‐pantothenic acid (Vitamin B_5_). When pantolactone is mixed with the chiral tag (in this case, trifluoropropylene oxide [TFPO]), two diastereomeric complexes result: homochiral complexes, where the analyte and tag have the same Cahn‐Ingold‐Prelog nomenclature ((*R*) or (*S*)), and heterochiral complexes, where they have the opposite designation. These diastereomeric complexes have different moments of inertia and are resolved by MRR without spectral overlap, with their stereochemistry assigned by comparison to quantum chemical calculations. In Figure 6, the transitions of the identified homochiral and heterochiral complexes are colored red and blue for clarity. In the negative‐going spectrum, where the analyte is racemic (equal amounts of (*R*) and (*S*)):the two complexes are both present at similar intensity. When enantiopure pantolactone is used, meanwhile, the heterochiral complex transitions increase in intensity while those of the homochiral complex decreases significantly, indicating that the heterochiral complex is formed at higher concentration. Because the chiral tag used in this measurement was known to have (*S*) stereochemistry, the pantolactone sample is therefore confirmed to be the (*R*) enantiomer—the desired form for synthesis of the bioactive pantothenic acid isomer.

In addition to the absolute configuration, the enantiomeric excess (ee) of a new compound can be determined using chiral tag rotational spectroscopy without the need for a reference sample. The response factors of the two diastereomeric complexes can be calibrated by measuring a sample with a racemic chiral tag mixture. The ee of the sample can be determined by measuring the relative signal levels of the diastereomeric complex in the measurements with racemic and enantiopure tag samples, and using the following equation to normalize the intensity of the complexes:

(5) $$N_{homochiral}=\frac{I_{enantiopure\;tag}}{I_{racemic\;tag}}$$

with a similar expression for deriving the normalized intensity of the heterochiral complex. From this, the abundance ratio of the two complexes in the enantiopure measurement can be determined using:

(6) $$R=\frac{N_{homochiral}}{N_{heterochiral}}$$

The ee of the analyte can finally be derived using:

(7) $$\frac{R+1}{R-1}=\left(ee_{tag}\right)\left(ee_{analyte}\right)$$

**FIGURE 7:**
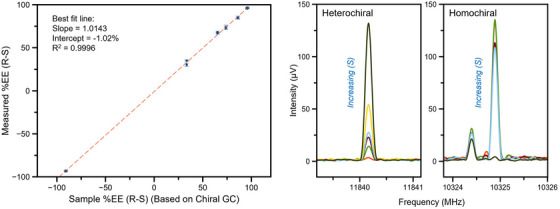
The enantiomeric excess of compounds can be determined using a targeted molecular rotational resonance (MRR) spectrometer. In these measurements, commercial samples of (R)‐ and (S)‐pantolactone are mixed to known purity levels and analyzed using MRR. In the left panel, the x‐axis represents the enantiomeric excess of each sample calculated using the enantiopurity of the chiral samples from chiral gas chromatography. Adapted with permission.^67^

The ee determination can be made using a single pair of transitions, one from each of the two complexes. For broadband measurements, where numerous transitions of each of the two complexes are simultaneously detected, these ee determinations can be averaged into a single value with improved precision. The quantitative accuracy of the ee values determined by MRR chiral tagging has been validated using gas chromatography (GC) and by the preparation of mixtures of the two enantiomers (Figure 7).^58^, ^67^

For implementation, broadband spectroscopy can be used to first determine the rotational transition frequencies for the most abundant chiral tag diastereomeric complexes formed using the racemic tag. Note that this initial spectroscopy measurement can use analyte samples of an ee—including racemic samples that are often available at lower cost. The subsequent measurements to determine the absolute configuration and ee of a test sample can be performed on a targeted MRR instrument in order to reduce the measurement time and the amount of sample required. In many applications, such as the development of new asymmetric reaction chemistry methods, measuring sample ee is of paramount importance and assigning the absolute configuration can occur later. Chiral tag ee measurements can be performed without requiring any computational chemistry analysis—which is only needed for establishing absolute configuration—allowing chemists to determine the selectivity of a new chemistry far more quickly.^76^

It should be noted that just as for chiral derivatization methods in NMR, the enantiopurity of the chiral tag is important as it affects the final population of the diastereomeric complexes (Equation (7)). The ee of each lot of the chiral tag is measured through a chiral tag measurement in an MRR spectrometer—either by complexation with another molecule of known enantiopurity, or in some cases by measuring its dimer, which also forms two diastereomeric complexes. This value is used in the ee analysis as described above. As long as the tag ee is known, it is not a strict requirement that the tag even be more enantiomerically pure than the analyte. If the tag ee were much lower than that of the analyte, then the precision of the derived ee value would decrease. However, the primary tags most often used in MRR—propylene oxide, TFPO and 1,1,1‐trifluoro‐2‐propanol—are all commercially available at ee > 99% so this is not currently a significant limitation.

### 3.4 Analysis of isotopic mixtures by MRR spectroscopy

MRR has unique capabilities for the complete analysis of mixtures of isotopically substituted compounds. As discussed in the Introduction, any change in molecular moments of inertia changes the characteristic rotational frequencies of a molecule. Even a single isotopic shift from ^12^C to ^13^C, or H to D, induces a measurable shift in the molecular rotational spectrum that is much greater than the instrument linewidth. In contrast to other analytical techniques, particularly MS and NMR, each isotopic variant (isotopologue and isotopomer) has a distinct, calculable, and well‐resolved chemical signature in the MRR spectrum, and so quantification of a mixture of isotopic species is straightforward. Once an accurate geometry of the analyte is known from computational chemistry, the MRR spectrum of any isotopically substituted variant can be predicted to excellent accuracy with a straightforward calculation.

There are a number of applications where isotopically labelled compounds are gaining increased interest, with a key emerging one being the development of deuterated pharmaceuticals. Due to the kinetic isotope effect, deuterated drugs are metabolized more slowly and so can extend the drug's half‐life or reduce the formation of unwanted metabolites.^77^ Two deuterated drugs, deutetrabenazine and deucravacitinib, have now been approved by the United States Food and Drug Administration for use.^78^ However, the kinetic isotope effect is strongly dependent on the position(s) of substitution. As a result, selective synthetic methods are required to incorporate isotopes precisely at the sites of interest, as are analytical techniques that can characterize the mixtures of isotopic species that can result. Particularly as the use of deuterated starting materials, as well as incorporation into early‐stage intermediates, may be practical methods for the selective preparation of deuterated pharmaceuticals, MRR can be a powerful tool for determining the isotopic species composition of these compounds.

In one example, a regio‐ and stereoselective method for the deuteration of benzene through the preparation of a tungsten complex was developed.^79^ Deuterated reagents were introduced at targeted points in the multi‐step synthesis to determine which isotope was incorporated at each position. MRR was used in this study to confirm that the stereoselectivity of the method was very high (with at least a 22:1 preference for the desired *cis*/*trans* isomer), and that the level of overdeuteration was very low. MRR has also been used in the characterization of a copper‐catalyzed hydrodeuteration chemistry to selectively add hydrogen and deuterium across a variety of alkene species. In the first study, the percentage of the desired isotopic species was greater than 94% across six analytes.^80^ The results of the MRR study were compared to ^1^H, ^2^H and ^13^C NMR spectroscopic data. In the NMR measurements, each observed resonance can be used to determine the total extent of deuterium incorporation at each position, but not the percentage of each isotopic variant in the sample. The MRR data were used to calculate the expected integrated NMR signal at each deuteration position, which matched the actual NMR data with excellent consistency.

This copper chemistry has also been used to prepare enantioisotopomers—molecules that are chiral by virtue of deuterium substitution—by incorporating a chiral ligand into the synthesis.^81^ In this case, the chiral tagging methodology is employed to allow enantioisotopomers to give resolved signals by MRR. With this approach, these compounds can be analyzed in the same way as any other chiral molecule, including determination of both the absolute configuration and ee without a reference sample. Despite the subtle difference in structure between the isotopomeric chiral tag complexes, the spectral shifts are much larger than the instrument linewidth—and the calculation of the chiral tag complex geometry is sufficient to allow the spectral patterns of each species to be accurately predicted. All of the other capabilities of chiral tagging MRR—including the accurate determination of ee, and the ability of targeted MRR instruments to perform routine ee measurements to support reaction optimization, are also extended to the analysis of enantioisotopomers.^76^

## 4 OUTLOOK AND FUTURE DIRECTIONS

The proceeding section described examples of applications where MRR can provide unique insight into solving challenging problems in analytical chemistry, with a focus on isomeric (or otherwise highly structurally related) compound resolution. As this is still a new technique for analytical chemists, the work performed over the last few years has unveiled two particularly important areas where development will be needed to allow for the routine use of MRR.

### 4.1 Future developments in spectral analysis

First, the process of fitting a rotational spectrum to determine the MRR parameters of the species that are present has, historically, been a highly manual process requiring extensive training. Even with good predictions of species from quantum chemistry, the high resolution of the spectrum can make the assignment process laborious, especially if the sample is in a mixture. In recent years, research groups have investigated a number of approaches to automating this fitting process, including automated double resonance experimental methods,^53^, ^82^ a brute‐force algorithm called Autofit to search for and identify any fits near an input guess geometry or within a specified rotational constants range,^83^, ^84^, ^85^ a method based on identifying common spectral patterns,^86^, ^87^ and a neural network approach.^88^ All of these methods have shown promise for component identification in both pure samples and mixtures, and continued development of these efforts will be essential in allowing analytical chemists to exploit the full potential of MRR.

A second, and perhaps more difficult, spectral analysis challenge pertains to the question of determining the molecular identity of an unknown molecule once its parameters are experimentally known. Databases of the parameters of molecules that have been previously analyzed by MRR are one useful approach, with small databases already in use with a focus on molecules of astronomical and/or atmospheric interest.^89^, ^90^ Extending this database to molecules of broader chemical and pharmaceutical interest is needed, which could also be supplemented by pure theoretical calculations to enable new compounds to be identified without requiring prior experimental measurement.

### 4.2 Sampling development in MRR

As MRR is a gas‐phase technique, the sampling methodology is also of prime importance. Most MRR sampling measurements rely on thermal volatilization techniques to liberate analytes into the gas phase, which limits or precludes the analysis of thermally labile molecules or those with low vapour pressure. Laser ablation sources have been coupled to MRR, as described above, and have allowed for the analysis of a number of nonvolatile compounds. However, this method often requires large amounts of solid samples (about 1 g per rod in some measurements):and complex sample preparation. Alternative sampling techniques that can easily analyze small amounts of crude samples, including oils and solutions, will be of extremely high value for accessing more varied and complex molecular classes. As described above, larger molecules also generally have lower sensitivity due to the increase in the rotational partition function, and so optimizing the technique sensitivity for larger molecular weight analytes is critical.

A few studies have also demonstrated the potential for coupling MRR spectroscopy with gas chromatography to provide interesting additional new capabilities for mixture resolution. In particular, isotopic mixtures have been analyzed showing that completely co‐eluting peaks can readily be distinguished and quantified by MRR using the methodology described in this review.^91^, ^92^ Other types of co‐eluting compounds can be resolved in the same way. At the same time, by providing temporal separation between components, mixtures with many components could be analyzed without the analysis challenges of extracting numerous spectral components from a rich MRR spectrum.

## 5 CONCLUSION

This review summarizes illustrative examples where MRR spectroscopy has been applied in recent years in the resolution and quantification of isomeric compounds. While the theoretical basis and core instrumentation capabilities for MRR are well developed, application areas will doubtless continue to emerge and will need to be supported by additional progress in the practical aspects described above. With this continued development, the use of MRR in analytical chemistry should continue to see significant expansion.

## CONFLICT OF INTEREST STATEMENT

Justin L. Neill and Brooks H. Pate have financial interests in BrightSpec, Inc., which is developing commercial instrumentation for MRR spectroscopy.

## Data Availability

Data sharing not applicable to this article as no datasets were generated or analyzed during the current study.
